# Burnout syndrome in Cypriot physiotherapists: a national survey

**DOI:** 10.1186/1472-6963-10-63

**Published:** 2010-03-11

**Authors:** Andreas Pavlakis, Vasilios Raftopoulos, Mamas Theodorou

**Affiliations:** 1 Open University of Cyprus, Healthcare Management Program, Nicosia, Cyprus; 2 Cyprus University of Technology, Nursing Department, Head of the Mediterranean Research Centre for Public Health and Quality of Care, Nicosia, Cyprus

## Abstract

**Background:**

Burnout in the healthcare workers is formally defined as a state of physical, emotional and mental exhaustion caused by long-term involvement in situations that are emotionally demanding.

**Methods:**

Using a random stratified sampling method and taking into account geographical location, specialty and type of employment, 172 physiotherapists working both in the private and public sectors completed an anonymous questionnaire that included several aspects related to burnout; the MBI scale, questions related to occupational stress, and questions pertaining to self image.

**Results:**

Almost half (46%) of the 172 participants believed that their job is stressful. Approximately 57% of the physiotherapists who worked in the public sector and 40% of those who worked in the private sector (p = 0.038) reported that their job is stressful. In total, 21.1% of participants met Maslach's criteria for burnout. The point prevalence of burnout was as follows: (1) 13.8% of those who worked in the public sector and 25.5% of those in the private sector (2) 22.2% of males and 20% of females (3) 21.6% who were married, 18% who were single and 33.3% who were separated. Gender was found to be associated with the level of personal accomplishment (chi-squared test; p = 0.049), as 17.8% of men compared with 24.3% of women reported high personal accomplishment. The number of years of working as a physiotherapist correlated negatively (r = -0.229, p = 0.004) with the total depersonalization score. Regression analysis showed that the perception that the job is stressful (p < 0.001) and the low salary (p = 0.016) were significant predictors of high emotional exhaustion scores, while age group (p = 0.027) predicted high scores of depersonalization and the employment sector (p = 0.050) as well as the low salary predicted high personal accomplishment scores.

**Conclusions:**

Burnout levels in physiotherapists in Cyprus ranged from low to moderate.

## Background

Cyprus is a new member of the European Union and has not yet introduced a National Health System. Therefore, the provision of health care services is based on public and private providers. The majority of the population is entitled to either free medical care, or to publicly provided medical care at reduced cost coverage, and the rest of the population purchases health services from the private sector. In addition, large companies and trade unions fund medical care for their employees and members respectively. The employees and the trade union members contribute according to their income.

According to the Webster's Medical Dictionary definition, the term burnout is characterised by "physical or emotional exhaustion, especially as a result of long-term stress or dissipation" [1]. The phenomenon has been extensively studied by Maslach & Jackson [2]. The onset of burnout is characterised by a gradual loss of idealism, energy, enterprise, and future aims resulting in emotional overload and exhaustion [3].

Herbert Freudenberger first coined the term "burnout syndrome" in 1974 when he described a set of symptoms of physical and mental exhaustion; primarily observed in healthcare workers [4]. He defined burnout syndrome as a state of mental and physical exhaustion caused by one's professional life [5]. The afflicted healthcare workers suffered from mood fluctuations, disturbed sleep and difficulties in concentration. Accompanying the mental distress were physical ailments such as backaches or digestive disorders.

Healthcare professionals work with others in emotionally demanding situations and are exposed to their clients' psychological, socioeconomic and physical problems. As a result, burnout can easily develop. Especially in sectors where long-term care is provided, burnout can become a factor of concern for the personnel [6]. In general, professionals experiencing burnout feel like they cannot contribute emotionally to the others as well as feeling disappointed by the day's caseload [7]. The professionals feel fatigue; they cannot provide basic care or develop a therapeutic relationship with their patients. Burnout was first detected in professionals providing psychiatric care to patients and similar instances of burnout were observed in critical care units, in units caring for terminally ill patients and in units caring for the chronically ill [8-10]. Research conducted among nursing educators indicates that burnout is associated with the demanding nature of the nursing profession, with the demanding working hours and shift-work as well as with feelings of professional inadequacy [11,12]. In a Northern Ireland research study it was concluded that most nursing educators develop burnout which is associated with feelings of personal dissatisfaction. Symptoms in men were more intense than in women [13].

Based on the wide research available, the burnout syndrome is more common in medical and nursing professionals without of course excluding other healthcare professionals such as physiotherapists, psychologists and social workers [14-16]. Despite this, the condition has not been investigated among physiotherapists in the same extent as for medical and especially nursing staff. A few studies conducted in Cyprus showed that the nursing staff of a regional hospital experience stress and melancholy to a large degree, nursing staff at the oncology centre smoke and use over the counter medication (without prescription) to a greater proportion than the general population and show higher levels of burnout [15,17]. In addition, other studies have shown that nurses in intensive care units suffer from high levels of stress and melancholy as well as experiencing burnout [18,19].

Physiotherapists, as well as occupational therapists are vulnerable to the onset of burnout because of their important role in the rehabilitation process that requires close interaction with the client. The literature concerning burnout in physiotherapists is sparse. Several studies have been reviewed: a 1993 study conducted in the Massachusetts hospitals showed that a large percentage of physiotherapists suffered from burnout syndrome [20], a 2002 study conducted in Japan [21] revealed that physiotherapists suffered from moderate burnout and a 2006 study conducted in Italy [22] concluded that physiotherapists suffered from high levels of professional exhaustion compared to nursing staff and physicians.

Taking the above into consideration and keeping in mind that the phenomenon of burnout has not been examined within the population of physiotherapists in Cyprus, a research study was undertaken to investigate the burnout syndrome within this population.

### Purpose

The purpose of the study was to explore the factors associated with burnout syndrome in Cypriot physiotherapists who work in the private and public healthcare sectors.

#### Study research questions

The research questions investigated were the following:

1. What is the point prevalence of burnout syndrome in Cypriot physiotherapists?

2. Which factors are associated with burnout syndrome in Cypriot physiotherapists?

3. What is the difference in burnout syndrome between Cypriot physiotherapists working in the private and the public sector?

## Methods

Subjects who were willing to participate and who met the following inclusion criteria were selected to participate in the study: 1) 18 years of age and older, 2) ability to speak and read Greek, and 3) being a registered physiotherapist, working in the public or private sector. Potential participants were recruited on the basis of their availability. They were approached by the researchers and were given a brief explanation of the purpose and aim of the study. An informed consent was obtained from those who agreed to participate and they were asked to complete the questionnaire. In Cyprus there are 383 physiotherapists working in the public and private sector. Based on calculations (95% CI and 5.5% margin of error) the sample size needed was 174 persons. Thus by using a random stratified sampling method and taking into account geographical location, specialty and type of employment, 200 physiotherapists working both in the private and public sectors were approached. One hundred and seventy five questionnaires were completed and returned, of which three were deemed incomplete and therefore not included in the study. The final sample consisted of 172 physiotherapists.

The Maslach & Jackson Burnout Inventory (MBI) is a 22-item instrument that assesses the degree of burnout in terms of three subscales: emotional exhaustion (EE), feelings of being emotionally exhausted and lack of energy, depersonalization (DP), feelings of impersonal response towards recipients of the service and lack of personal accomplishment (PA), feeling of incompetence [23]. This instrument has been validated in the Greek language by Anagnostopoulos et al. and has been recently tested on a sample of oncology nurses and physicians [24,25]. High scores in the EE or DP scales or low scores in the personal accomplishment scale indicate high levels of burnout.

The questionnaire was divided into two main parts. The first contained demographic information, questions related to the level of occupational stress, and the self image of the Cypriot physiotherapist. The second part included the MBI scale.

### Ethical issues

The Healthcare Management Program of the Open University of Cyprus, acting as an ethics committee, granted written ethical approval for this study. Permission to carry out the study in the healthcare settings was provided by the Director of the Medical Services and Public Health of the Cyprus Ministry of Health. The Cyprus Association of Physiotherapists supported the integrity of the study and helped in the dissemination of the questionnaires among its members.

### Data analysis

All of the items were coded and scored, and the completed questionnaires were included in the data analysis set. SPSS-15 (SPSS, Chicago, IL, USA) was used to analyze the data. The chi-square test was used to explore the existence of a statistically significant relationship between the categorical variables. The t-test was used to assess whether the means of two groups were statistically different from each other. P values < 0.05 were considered to be statistically significant, unless otherwise stated. Internal consistency of the MBI scale was assessed by calculating Cronbach's alpha. Multiple regression analysis has been used to explore the factors that are associated with levels of burnout. The equation included the three subscales (EE, PA, DP) overall score as dependent variable. Data collection was conducted between May and July of 2008.

## Results

One hundred and seventy two physiotherapists participated in the study (64% were female). As seen in Table 1, more than two-thirds of the respondents were above the age of 40; more than half were married and had two or more children (57.6%). Two-thirds of the respondents (64.2%) were employed in the private sector and approximately 50% were qualified physiotherapists. More than half of the respondents have worked in the field less than 10 years.

**Table 1:** Social and demographic characteristics of the sample

Variable	N	%
**Gender**		
Men	62	36
Women	110	64
**Age**		
<30 yrs	13	7.9
31 - 40 yrs	17	10.4
41 - 50 yrs	50	30.5
51 - 60 yrs	71	43.3
>60 yrs	13	7.9
**Marital status**		
Married	104	63.8
Single	51	31.3
Divorced/Separated	8	4.9

### Self-image of the Cypriot physiotherapist

The participants were asked to rate themselves as professionals "how would you score yourself as a professional?" by using a scale that ranged from 0 to 10. The mean score was 7.97 ± 1.08 ranging from 6 to 10. Additionally, they were asked to rate the status of the Cypriot physiotherapist, in general. The subsequent mean score was 7.36 ± 1.33, ranging from 3 to 10. The t-test revealed a statistically significant difference (p = 0.015) of men (n = 44, 6.95 ± 1.59) and women (n = 107, 7.53 ± 1.17) in the mean score in the above question.

### Characteristics of burnout syndrome in Cypriot physiotherapists

Almost half (46%) of the participants believed that their job was stressful. Around 57% of the physiotherapists who worked in the public sector as opposed to 40% of those who worked in the private sector (p = 0.038) reported that their job was stressful.

Table 2 summarizes the mean scores computed for each of the three MBI subscales (EE, DP, PA). Eight percent of the participants were in the high EE range, 23% scored high in the PA section and 17.4% scored high in the DP section of the scale.

**Table 2:** Mean scores in the MBI subscales

MBI subscale	Mean ± sd	Low (95%CI)	Moderate (95%CI)	High (95%CI)
EE	16.55 ± 9.09	69.60 (62.38-76.74)	22.42 (15.85-28.86)	8.15 (3.82-12.32)
PA	5.20 ± 4.61	42.23 (34.52-49.94)	34.83 (27.34-42.21)	23,01 (16.41-29.54)
DP	39.50 ± 5.58	59.02 (51.32-66.68)	23.60 (16.97-30.23)	17.39 (11.47-23.30)

The 95%CI have been calculated using the binomial Wald assumption

EE: high (≥ 31), moderate (21-30), low (≤ 20)

PA: high (≤ 35), moderate (41-36), low (≥ 42)

DP: high (≥ 11), moderate (6-10), low (≤ 5)

In total, 21.1% of participants met Maslach's criteria for burnout [a high DP score (>11) and/or high EE score (>31)]. The point prevalence of burnout was: (1) 13.8% of those who work in the public sector and 25.5% in the private sector (2) 22.2% were males and 20% were the females (3) 21.6% were married, 18% single and 33.3% separated. The factor that correlated with burnout onset was therapists' belief that their job was stressful as chi-squared (p = 0.049) showed that 28.4% of those who believed that their job was stressful suffered from burnout as opposed to the 15.5% of those who did not feel that their job was stressful. Although the t-test did not reveal a statistically significant difference in the mean MBI subscales' scores between physiotherapists who work in the private and those in the public sectors, the chi-squared test (p = 0.011) showed that more physiotherapists from the private sector experienced high EE as opposed to those in the public sector (11.8% compared to 1.7%), while the opposite was the case with moderate EE (16.7% versus 32.8%). Thirty six percent of the physiotherapists worked in the pediatric sector. Chi-squared test was statistically significant (p = 0.034) and revealed that 15.5% of the pediatric physiotherapists reported feelings of high EE compared to 3.9% of non-pediatric physiotherapists.

Table 3 presents the MBI subscale scores by social and demographic characteristics. Age group correlated with DP mean score (r = -0.243, p = 0.002). Mean EE score was significantly higher in women (p = 0.018), while mean PA score was significantly higher in men (p = 0.022). Gender, however, was not a significant variable for DP. Marital status and age group did not correlate with MBI dimensions. The employment sector affected the level of DP (p = 0.034) as 13.8% of those who worked in the public sector compared to 19.4% of those who worked in the private sector were in the high DP range.

**Table 3:** The associations between variables and the three burnout subscales (mean scores) explored using t-test and ANOVA tests as appropriate

Variable	EE mean (95% CI)	DP mean (95% CI)	PA mean (95% CI)
**Gender**			
Men	13.82 (11.19-16.45)	5.56 (4.10-7.02)	41.09 (39.59-42.59)
Women	17.58 (15.91-19.26)	4.97 (4.15-5.80)	38.97 (37.93-40.01)
p value	0.018^2^	0.488	0.022
			
**Marital status**			
Married	17.13 (15.30-18.95)	4.88 (3.97-5.80)	39.38 (38.23-40.53)
Single	15.76 (13.47-18.05)	5.74 (4.53-6.95)	39.38 (37.97-40.79)
Divorced/Separated	14.33 (1.80-30.46)	4.50 (1.46-10.46)	44.33 (41.17-47.49)
p value	0.572^1^	0.522	0.100
			
**Employment sector**			
Public	18.22 (15.04-19.17)	4.84 (3.67-6.16)	39.28 (37.60-40.88)
Private	15.61 (14.38-18.22)	5.40 (4.42-6.21)	39.62 (38.63-40.68)
p value	0.080^2^	0.467	0.711
			
**Job selection**			
Own decision	16.13 (14.53-12.73)	4.89 (4.13-5.65)	39.62 (38.71-40.54)
Others' empowerment	18.28 (15.12-21.44)	6.12 (3.93-8.31)	39.16 (36.29-42.03)
Others decided it	21.00 (2.70-44.70)	11.33 (9.90-12.77)	36.67 (24.41-48.92)
p value	0.387^1^	0.031	0.630
			
**My job is stressful**			
Yes	20.88 (18.85-22.91)	5.89 (4.78-7.00)	38.14 ± 5.52 (36.85-39.42)
No	12.85 (11.15-14.54)	4.58 (3.63-5.54)	40.74 ± 5.45 (39.55-41.92)
p value	<0.001^2^	0.077	0.003
			
**Salary perceived as low**			
Yes	17.85 (8.89-15.69)	5.49 (1.83-5.31)	38.9 (37.98-39.93)
No	12.29 (16.24-19.47)	3.57 (4.68-6.31)	42.67 (40.88-44.45)
p value	0.005^2^	0.048^2^	0.040^2^
			
**Lack of cooperation with colleagues**			
Yes	17.34 (12.84-17.97)	5.67 (4.77-6.58)	39.17 (38.10-40.24)
No	15.40 (15.58-19.10)	4.17 (2.95-5.40)	40.43 (38.86-41.99)
p value	0.216^2^	0.050^2^	0.187^2^
			
**Lack of support from the superiors**			
Yes	18.16 ± 9.52 (12.32-16.12)	5.47 (4.46-6.48)	38.72 (37.62-39.81)
No	14.22 ± 7.29 (16.11-20.22)	4.39 (3.31-5.47)	40.61 (39.09-42.13)
p value	0.006^2^	0.146^2^	0.046^2^
			
**Lack of opportunities for professional development**			
Yes	17.32 (8.98-16.13)	5.27 (4.47-6.06)	39.12 (38.17-40.07)
No	12.56 ± (15.74-18.90)	4.72 (2.57-6.88)	42.89 (41.17-44.61)
p value	0.017^2^	0.626^2^	0.006^2^

^1 ^ANOVA test ^2 ^T-test analysis

Furthermore, mean DP score was higher in those who replied that they were influenced by others in selecting the profession of physiotherapist but the difference was not statistically significant. The mean EE score was significantly higher in the physiotherapists who believed that their job was stressful. Also, the mean PA score was lower in those who believed their job was a stressful one.

Chi-square test revealed that gender correlated with the level of PA (p = 0.049) as 17.8% of men vs 24.3% of women were in the high PA range. The number of years working as a physiotherapist correlated negatively (r = -0.229, p = 0.004) with total DP scores, which means that the participants with a lot of experience had lower feelings of DP.

Of those who believed that their job was stressful, 14.9% scored high on the EE subscale and 31.1% of them scored low in the PA subscale. Of those who believed that their job was not stressful, 2.4% scored high on the EE subscale and 16.7% scored low in the PA subscale (p < 0.001).

The ANOVA test showed that those in the high EE range rated (8.77 ± 0.92) the social status of the Cypriot physiotherapist higher compared to those in the moderate range (7.85 ± 1.10) and those in the low range (7.90 ± 1.07).

Pediatric physiotherapists experienced higher levels of EE feelings at (15.5%) than non-pediatric physiotherapists (3.9%) with a statistical significance of p = 0.034.

To further explore organizational factors that are associated with the onset of burnout, participants were asked to answer whether they spent time thinking about their salary, the lack of cooperation with their colleagues, the lack of cooperation with their superiors, and the lack of opportunities for professional development. The vast majority of the participants (85.5%) felt that they were underpaid, while 70.3% commented on the lack of cooperation with their colleagues; 59.2% felt a lack of support from their superiors and 88.2% pointed out the lack of opportunities for professional development. As seen in Table 3, participants who obtained higher scores of EE commented the low salary, the lack of support from the superiors and the lack of opportunities for professional development. Those with high DP scores were disappointed with the low salary and the lack of collaboration and communication with their colleagues. Physiotherapists who scored low in the PA subscale made negative comments regarding the low salary, the lack of cooperation they have with their superiors and the lack of opportunities for professional development.

### Factors associated with high scores of burnout

Coefficient estimates, level of significance, and explained variance are presented in Table 4. Multiple regression analysis revealed that the predictors of: (1) EE were the perception of a stressful job (p < 0.001) and the low salary (p = 0.016) but the model was not significant, (2) DP was age group (p = 0.027) but the model was not significant and (3) PA were the employment sector (p = 0.050), the perception of a stressful job (p = 0.005) and the low salary (p = 0.007), the model was significant.

**Table 4:** Regression analysis by using mean score of the three burnout subscales as dependent variable

Variable	EE		DP		PA	
	**Beta**	**Sig**	**Beta**	**Sig**	**Beta**	**Sig**
Intercept	5.93	0.458	2.502	0.555	51.681	<0.001
Gender	1.794	0.321	-0.098	0.919	-1.479	0.199
Age group	0.889	0.246	-0.902	0.027	0.295	0.537
Marital status	-0.822	0.557	0.463	0.532	1.463	0.097
Employment sector	1.840	0.244	2.502	0.990	-1.969	0.050
Job selection	1.609	0.319	1.675	0.055	-1.006	0.328
My job is stressful	-7.551	<0.001	-0.973	0.201	2.536	0.005
Salary perceived as low	5.309	0.016	0.986	0.404	-3.823	0.007
Lack of cooperation with colleagues	0.049	0.976	1.355	0.172	0.003	0.998
Lack of support from the superiors	3.177	0.076	0.121	0.695	-0.754	0.502
Lack of opportunities for professional development	2.101	0.823	-0.536	0.899	-1.813	0.264

R^2 ^change	25.2%		11.9%		19%	

### Reliability analysis

Internal consistency of the subscales proved good as Cronbach's alpha was 0.84 for the emotional exhaustion subscale, 0.62 for the lack of personal accomplishment and 0.58 for the depersonalization [26].

## Discussion

Our research has explored the factors associated with physiotherapists' burnout. The strength of the study was its representativeness, since 45% of all the physiotherapists who work in Cyprus were surveyed. To our knowledge, this is the first published nationwide research in the field in Cyprus and could be used for the development of a national action plan for the prevention and management of burnout in Cypriot physiotherapists.

In the current research the reliability analysis revealed that Cronbach's alpha was 0.62 for the PA and 0.58 for the DP scale. In the validation study of the Greek version of the MBI that was conducted by Anagnostopoulos and Papadatou revealed the followings: EE alpha = 0.84, PA alpha = 0.71 and depersonalization alpha = 0.55 [27]. In a similar research in a sample of nurses in Greece: EE alpha = 0.81, PA alpha = 0.78 and DP alpha = 0.67 [12]. In another research that we have conducted in oncology nurses in Greece: EE alpha = 0.85, PA alpha = 0.78 and DP alpha = 0.70 [25]. In Soler et al. study Cronbach's alpha for depersonalization ranged across the participant countries from 0.46 to 0.91 and for personal accomplishment from 0.67 to 0.85 [15]. Cronbach's alpha depends on the number of scale items and on the consistency the participants respond to the items of the scale. A further research could be useful to test the reliability into other samples.

The analysis showed that burnout levels in physiotherapists ranged from low to moderate. The findings of the current study share similarities with others, as the majority of the related studies attributed the point prevalence of burnout in physiotherapists to their direct involvement with disabled individuals [12,21,22,28,29]. Additionally, working with younger patients and particularly with children tends to be more distressing; three times more pediatric physiotherapists reported feelings of high emotional exhaustion in comparison with non-pediatric physiotherapists [30]. The burden of disability is heavier in younger patients.

The percentage of physiotherapists with high EE was lower in this research compared to the findings of other studies [20,29]. According to Maslach et al, the emotional exhaustion subscale reflects mainly the organizational and the social climate of the work environment [23]. Consequently, Cypriot physiotherapists showed moderate feelings of EE perhaps because of their adaptation to the climate of their organization. They have the capacity to learn new skills related to their job and to adapt their expectations about their work environment according to their experience (they know what to expect and have lower expectations, a protective mechanism that leads to higher levels of emotional exhaustion). This can be concluded from the fact that 23.3% of the participants indicated high levels of PA that is indicative of how well the physiotherapist thinks he is performing at work and whether his/her work is acknowledged by others [22]. In support of the previous finding, perceived job-related stress influenced the level of EE and PA but not DP [31]. Another explanation could be that several socio-demographics, cultural differences, personality traits and attitudes influence the onset of emotional exhaustion [21]. A further cross-cultural research explaining these factors would be useful to clear this point.

It was expected that the employment sector (private vs. public) would affect burnout levels because physiotherapists who work in the private sector are more autonomous and better paid than those who are employed in the public sector. Physiotherapist who work in the private sector (self-employed or as an employee) reported higher feelings of EE and burnout than their colleagues in the public healthcare settings. A possible reason for this could be the uncertainty about job security in the private sector which is influenced by changes in the external environment such as changes in the economy, the regulations with the Insurance Organizations and the social climate. Additionally private physiotherapists have a closer and ongoing relationship with their clients, an increased workload and exposure to the emotional and physical needs of their patients. Furthermore, the physiotherapists who work in the hospital usually have contact with their patients during the acute phase of their illness as opposed to the private sector physiotherapists who have a longer collaboration and develop closer relationships.

Those who replied that a "significant other" influenced their decision to follow the profession of physiotherapist scored higher in DP. It seems that these individuals are less self- directed and as a result take others' opinions regarding their work into serious consideration. These individuals may give a lot more emphasis to the feedback from patients and from their colleagues. Negative feedback may impact their perception of their achievement. They may have lower self esteem that might result in diminished appraisal of their professional worth [32]. In a recent Greek research study, higher levels of DP and EE were associated with lower self-esteem in nurses [12].

Although gender did not predict burnout prevalence, it correlated with EE and PA, with females being more susceptible [29]. Perhaps the factor that explains this phenomenon is that women often have a double role, namely the one of the healthcare professional and the other of the mother; this causes them more stress and drains their energy overall [32].

The majority of the participants replied that they were underpaid; they did, however, have enough support from their supervisors and from their colleagues [29,33]. The physiotherapists who felt that they were underpaid scored higher on all three burnout subscales, which means that salary-satisfaction is a major contributing factor of burnout in their case, as it may affect their social image and their job satisfaction, which in turn are predominant factors in professional burnout [12]. Future studies should examine certain factors that correlate with burnout; examples could include self-esteem, organizational and work related factors.

### Limitations

A limitation of the study was that the survey was conducted among the physiotherapists who are registered with the Cyprus Association of Physiotherapists. Furthermore, only Greek-speaking individuals were approached. Another limitation is the poor reliability of the two subscales of burnout (PA and DP).

## Conclusions

Burnout is a common problem in physiotherapists in Cyprus with high levels affecting one-fifth of the participants of this study. In all 8%, 17.4% and 23% of the participants reported high levels of EE, DP and PA, respectively. High burnout scores are more likely in association with certain variables used in the current study such as: low salary, employment sector, age group and job-related stress. Further research is needed to explore the problem in depth as well as to develop intervention programs that are effective.

## Competing interests

The authors declare that they have no competing interests.

## Authors' contributions

VR wrote the manuscript, analysed the data, interpreted the results, revised the manuscript and coordinated the study, AP collected the data and contributed to the writing of the background, MT was involved in the study design.

## Pre-publication history

The pre-publication history for this paper can be accessed here:

http://www.biomedcentral.com/1472-6963/10/63/prepub
